# Supplementary material for: Data processing choices can affect findings in differential methylation analyses: an investigation using data from the LIMIT RCT
Source: PeerJ. 2023 Feb 3;11:e14786. doi: 10.7717/peerj.14786 (PMC9901304; doi:10.7717/peerj.14786)
Supplement: Supplemental Information 1 [file peerj-11-14786-s001.docx]

Supplementary Information

**Table S1. Baseline Characteristics of LIMIT participants with Cord Blood DNAm Data**

| **Characteristic** | **Lifestyle Advice** | **Standard Care** | **Overall** |
| --- | --- | --- | --- |
| Overall Numbers | n=325 | n=320 | n=645 |
| BMI (kg/m2): Median (IQR) | 31.40 (28.10, 36.20) | 31.45 (27.98, 36.90) | 31.40 (28.00, 36.50) |
| BMI Category: (N%) |  |  |  |
| - 25.0-29.9 | 129 (39.69) | 130 (40.62) | 259 (40.16) |
| - 30.0-34.9 | 99 (30.46) | 86 (26.88) | 185 (28.68) |
| - 35.0-39.9 | 58 (17.85) | 55 (17.19) | 113 (17.52) |
| - ≥40.0 | 39 (12.00) | 49 (15.31) | 88 (13.64) |
| Height(cm): Mean (SD) | 165.29 (6.66) | 164.73 (6.48) | 165.01 (6.57) |
| Weight(kg): Mean (SD) | 89.81 (17.48) | 89.75 (18.65) | 89.78 (18.06) |
| Parity: N(%) |  |  |  |
| - 0 | 141 (43.38) | 128 (40.00) | 269 (41.71) |
| - 1+ | 184 (56.62) | 192 (60.00) | 376 (58.29) |
| Age at TE: Mean (SD) | 29.28 (5.56) | 29.63 (5.24) | 29.45 (5.41) |
| Smoking: N(%) |  |  |  |
| - Yes | 274 (84.31) | 274 (85.62) | 548 (84.96) |
| - No | 47 (14.46) | 37 (11.56) | 84 (13.02) |
| - Missing | 4 (1.23) | 9 (2.81) | 13 (2.02) |
| Ethnicity: N(%) |  |  |  |
| - Non-Caucasian | 29 (8.92) | 29 (9.06) | 58 (8.99) |
| - Caucasian | 294 (90.46) | 291 (90.94) | 585 (90.70) |
| - Missing | 2 (0.62) | 0 (0.00) | 2 (0.31) |
| Quintile of Relative Socioeconomic Disadvantage: N(%) |  |  |  |
| - Q1 | 107 (32.92) | 87 (27.19) | 194 (30.08) |
| - Q2 | 61 (18.77) | 83 (25.94) | 144 (22.33) |
| - Q3 | 59 (18.15) | 52 (16.25) | 111 (17.21) |
| - Q4 | 46 (14.15) | 52 (16.25) | 98 (15.19) |
| - Q5 | 52 (16.00) | 46 (14.37) | 98 (15.19) |
| Infant Sex: N(%) |  |  |  |
| - Male | 164 (50.46) | 163 (50.94) | 327 (50.70) |
| - Female | 161 (49.54) | 157 (49.06) | 318 (49.30) |
| Study Site: N(%) |  |  |  |
| - WCH | 135 (41.54) | 136 (42.50) | 271 (42.02) |
| - FMC | 98 (30.15) | 103 (32.19) | 201 (31.16) |
| - LMH | 92 (28.31) | 81 (25.31) | 173 (26.82) |

**Table S2. Numbers assigned to fake groups**

|  | **Long** | **Short** | **Total** |
| --- | --- | --- | --- |
| **Tabby** | 134 | 192 | 326 (50.5) |
| **Tortoiseshell** | 126 | 193 | 319 (49.5) |
| **Total** | 260 (40.3) | 385 (59.7) | 645 |

Figure S1. UpSet plot showing overlap between models in the DMPs with p value <1e-100 for Infant Sex


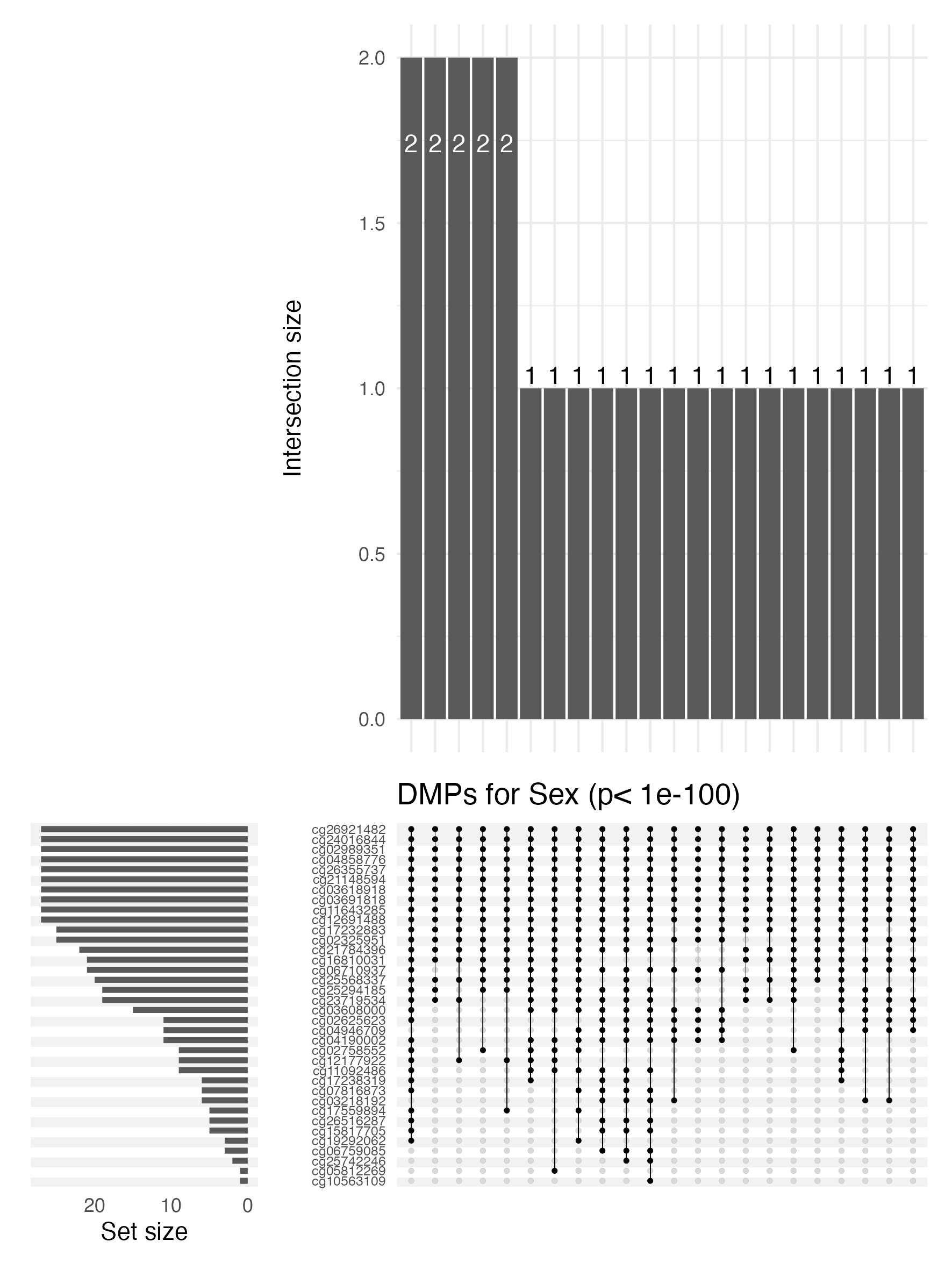


**Table S3 RefGene Names and Accession Numbers for DMPs (p<1e-100) for Infant Sex**

| **Chr** | **Name** | **UCSC RefGene Name** | **UCSC RefGene Accession** |
| --- | --- | --- | --- |
| chr1 | cg03618918 |  |  |
| chr1 | cg12177922 | HAX1 | NM_001018837;NM_006118 |
| chr1 | cg12691488 |  |  |
| chr1 | cg15817705 |  |  |
| chr1 | cg24016844 | C1orf103 | NM_001006945;NM_018372 |
| chr1 | cg25742246 |  |  |
| chr11 | cg04858776 |  |  |
| chr11 | cg17232883 |  |  |
| chr11 | cg25294185 | RNASEH2C | NM_032193 |
| chr12 | cg03691818 | KRT77 | NM_175078 |
| chr12 | cg07816873 | ERC1 | NM_178039;NM_178040;NR_027948;NM;  NR_027949;NM_178039;NR_027946 |
| chr13 | cg02625623 |  |  |
| chr13 | cg06710937 |  |  |
| chr13 | cg26355737 | TFDP1 | NR_026580;NM_007111 |
| chr14 | cg02325951 | FOXN3 | NM_005197;NM_001085471 |
| chr14 | cg21148594 |  |  |
| chr15 | cg23719534 |  |  |
| chr16 | cg04946709 | LOC644649 | NR_028471 |
| chr16 | cg26921482 | AMDHD2 | NM_001145815;NM_015944 |
| chr17 | cg03218192 | AP2B1 | NM_001282;NM_001030006 |
| chr17 | cg16810031 | ZPBP2 | NM_199321;NM_198844 |
| chr18 | cg05812269 | RIOK3 | NM_003831 |
| chr19 | cg03608000 | ZNF69 | NM_021915 |
| chr2 | cg02989351 | YWHAQ | NM_006826 |
| chr2 | cg06759085 | NAB1 | NM_005966 |
| chr20 | cg19292062 | CSNK2A1 | NM_177560;NM_001895;NM_177559;NM_001895 |
| chr22 | cg04190002 | SHANK3 | NM_001080420 |
| chr3 | cg02758552 | GPX1 | NM_000581;NM_201397 |
| chr3 | cg11643285 | RFTN1 | NM_015150 |
| chr3 | cg17238319 | RFTN1 | NM_015150 |
| chr6 | cg10563109 |  |  |
| chr6 | cg11092486 |  |  |
| chr6 | cg25568337 | ARID1B | NM_175863;NM_017519;NM_020732 |
| chr7 | cg17559894 |  |  |
| chr7 | cg21784396 | PRRT4 | NM_001114726 |
| chr7 | cg26516287 | SCIN | NM_001112706;NM_033128 |

Figure S2 UpSet plot showing overlap between models in DMPs with p value <0.05 for effect of intervention at the mean cohort BMI value


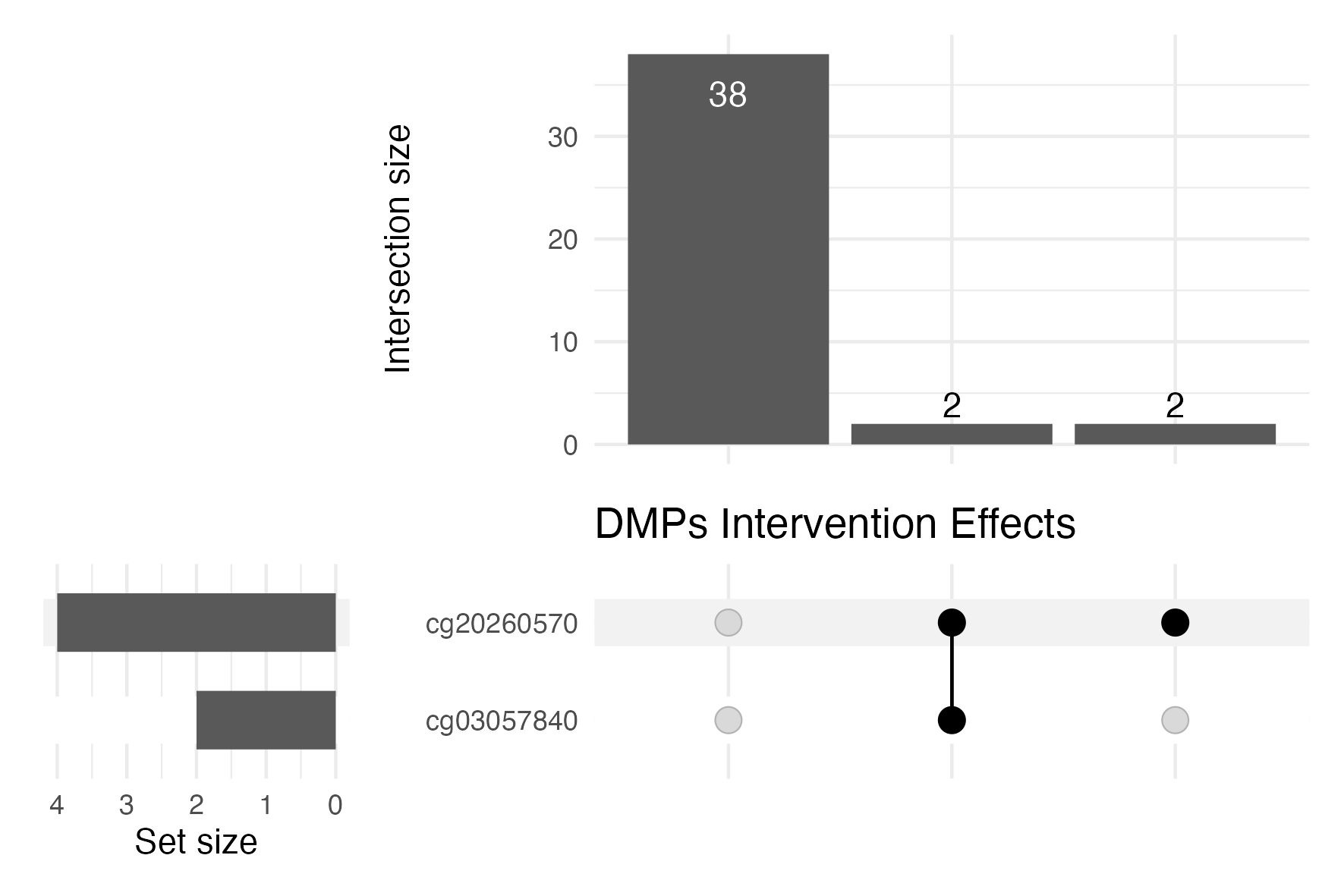


Figure S3 UpSet plot showing overlap between models in DMPs with p value <0.01 for effect of BMI in the Standard Care group


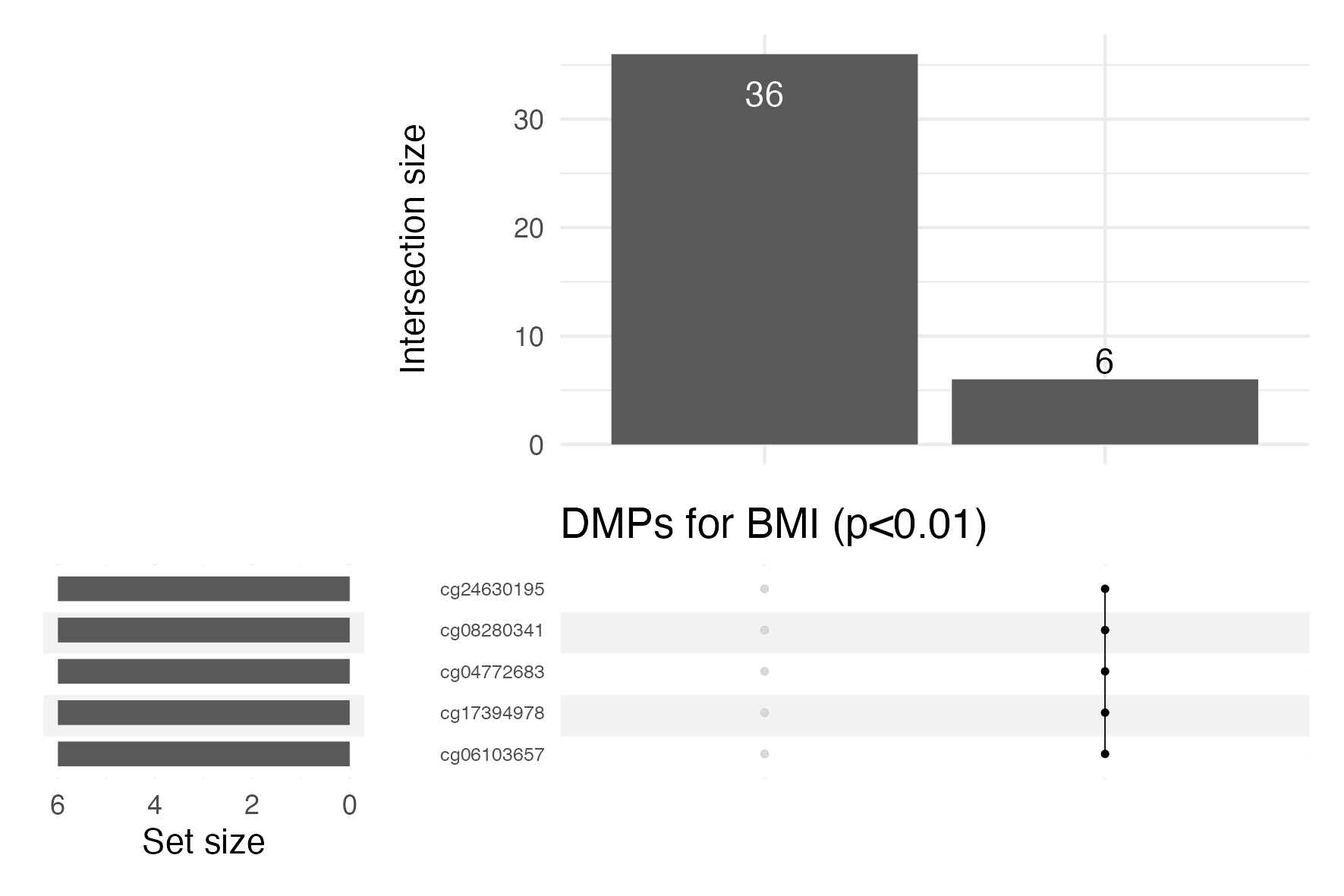


**Figure S4 UpSet plot showing overlap between models in DMPs with p value < 0.01 for Short-Haired in Tabby**


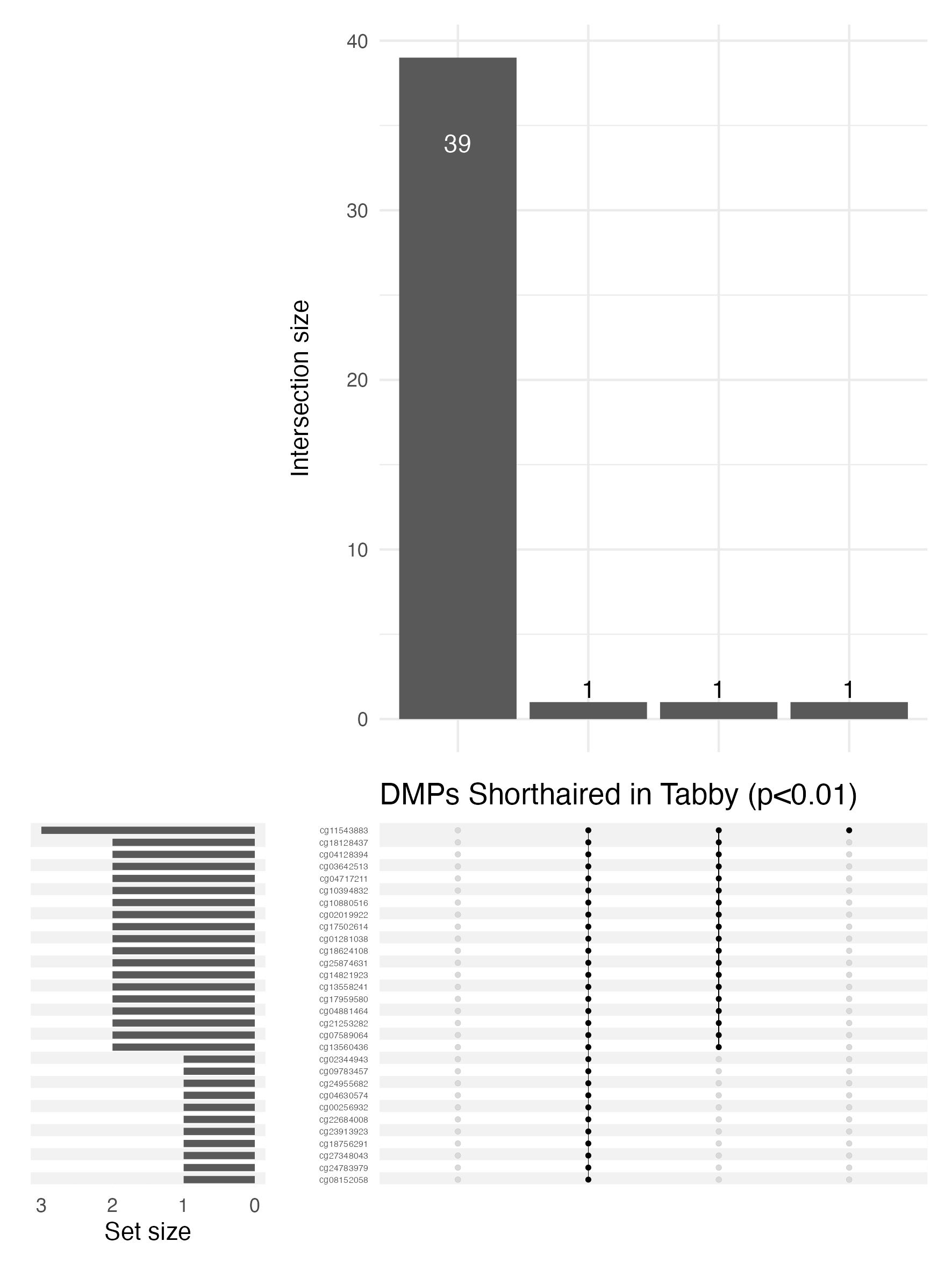


Table S4 Models and Contrast Matrices

| Model | Model Matrix | Contrasts |  |
| --- | --- | --- | --- |
| Fake Group and Infant Sex | | | |
| Fake Groups + infant sex:  Model 1 (unadjusted) | ~ fakegrp*fakegrp2 + BSex  $\beta_{0}+ \beta_{1}FakeGroup1+ \beta_{2}FakeGroup2+ \beta_{3}Sex+ \beta_{4}FakeGroup1\times FakeGroup2$ | Effect of Tortoiseshell (vs Tabby) in Short-Haired and in Long-Haired  c(0, 1, 0, 0, 0)  c(0, 1, 0, 0, 1)  Effect of Long-Haired (vs Short-Haired) in Tabby and in Tortoiseshell:  c(0, 0, 1, 0, 0)  c(0, 0, 1, 0, 1)  Effect of infant sex  c(0, 0, 0, 1, 0) |  |
| Fake Groups:  Model 2  (adjustment for 55 batches) | ~ fakegrp*fakegrp2 + Bsex + factor(Sentrix_Barcode)  $\beta_{0}+ \beta_{1}FakeGroup1+ \beta_{2}FakeGroup2+ \beta_{3}Sex+ \beta_{4}Batch_{2}+ . . . +\beta_{57}Batch55 + \beta_{58}FakeGroup1\times FakeGroup2$ | Effect of Tortoiseshell (vs Tabby) in Short-Haired and in Long-Haired  c(0, 1, rep(0, 57))  c(0, 1, rep(0, 56), 1)  Effect of Long-Haired (vs Short-Haired) in Tabby and in Tortoiseshell:  c(0, 0, 1, rep(0, 56))  c(0, 0, 1, rep(0, 55), 1)  Effect of infant sex  c(0, 0, 0, 1, rep(0, 55)) |  |
| Fake Groups:  Model 3  (adjustment for batch and cell type) | ~ fakegrp1*fakegrp2 + Bsex + factor(Sentrix_Barcode) + Bcell + CD4T + CD8T + Gran + Mono + NK + nRBC  $\beta_{0}+ \beta_{1}FakeGroup1+ \beta_{2}FakeGroup2+ \beta_{3}Sex+ \beta_{4}Batch_{2}+ . . . +\beta_{57}Batch55 +\beta_{58}Bcell+ \beta_{59}CD4T+ \beta_{60}CD8T+ \beta_{61}Gran+ \beta_{62}Mono+ \beta_{63}NK+ \beta_{64}nRBC+ \beta_{65}FakeGroup1\times FakeGroup2$ | Effect of Tortoiseshell (vs Tabby) in Short-Haired and in Long-Haired  c(0, 1, rep(0, 64))  c(0, 1, rep(0, 63), 1)  Effect of Long-Haired (vs Short-Haired) in Tabby and in Tortoiseshell:  c(0, 0, 1, rep(0, 63))  c(0, 0, 1, rep(0, 62), 1)  Effect of infant sex  c(0, 0, 0, 1, rep(0, 62)) |  |
| Intervention(Treatment) and BMI Effects | | | |
| BMI + Intervention: Model 1 (unadjusted) | ~ Treatment*bmic  $\beta_{0}+\beta_{1}Intervention+ \beta_{2}BMI_{c}+ \beta_{3}Intervention\times BMI_{c}$ | Effect of intervention at mean BMI and at +5 BMI:  c(0, 1, 0, 0)  c(0, 1, 0, 5)  Effect of 5 kg/m^2^ increase in BMI in standard care group and in lifestyle group  c(0, 0, 5, 0)  c(0, 0, 5, 5) |  |
| BMI + Intervention: Model 2 (adjusted for 55 batches) | ~ Treatment*bmic + factor(Sentrix_Barcode)  $\beta_{0}+\beta_{1}Intervention+ \beta_{2}BMI_{c}+ {\beta_{3}Batch_{2}. . . \beta_{56}Batch_{55}+ \beta}_{57}Intervention\times BMI_{c}$ | Effect of intervention at mean BMI at at +5 BMI:  c(0, 1, rep(0, 56))  c(0, 1, rep(0, 55), 5)  Effect of 5kg/m^2^ increase in BMI in standard care group and in lifestyle group  c(0, 0, 5, rep(0, 55))  c(0, 0, 5, rep(0, 54), 5)) |  |
| BMI + Intervention: Model 3 (adjusted for batch and cell type proportion) | ~ Treatment*bmic + factor(Sentrix_Barcode) + Bcell + CD4T + CD8T + Gran + Mono + NK + nRBC  $\beta_{0}+\beta_{1}Intervention+ \beta_{2}BMI_{c}+ {\beta_{3}Batch_{2}. . . \beta_{56}Batch_{55}+\beta_{56}Bcell+ \beta_{57}CD4T+ \beta_{58}CD8T+ \beta_{59}Gran+ \beta_{60}Mono+ \beta_{61}NK+ \beta_{62}nRBC+ \beta}_{63}Intervention\times BMI_{c}$ | Effect of intervention at mean BMI at at +5 BMI:  c(0, 1, rep(0, 63))  c(0, 1, rep(0, 62), 5)  Effect of 5kg/m^2^ increase in BMI in standard care group and in lifestyle group  c(0, 0, 5, rep(0, 62))  c(0, 0, 5, rep(0, 61), 5)) |  |

**Table S5 Spearman rank correlations for p value rankings: BMI in Standard Care**

Post= probe filtering after normalisation ; Pre=probe filtering before normalisation

Adj=batch included as covariate in the model; SCB=Supervised ComBat algorithm; UCB=Unsupervised ComBat algorithm

|  |  | **SQN** | | | | | | **BMIQ** | | | | | | **SWAN** | | | | | |
| --- | --- | --- | --- | --- | --- | --- | --- | --- | --- | --- | --- | --- | --- | --- | --- | --- | --- | --- | --- |
|  |  | **Post**  **Adj** | **Pre**  **Adj** | **Post**  **SCB** | **Pre**  **SCB** | **Post**  **UCB** | **Pre**  **UCB** | **Post**  **Adj** | **Pre**  **Adj** | **Post**  **SCB** | **Pre**  **SCB** | **Post**  **UCB** | **Pre**  **UCB** | **Post**  **Adj** | **Pre**  **Adj** | **Post**  **SCB** | **Pre**  **SCB** | **Post**  **UCB** | **Pre**  **UCB** |
| **SQN** | **Post Adj** | 1.00 |  |  |  |  |  |  |  |  |  |  |  |  |  |  |  |  |  |
|  | **Pre Adj** | 1.00 | 1.00 |  |  |  |  |  |  |  |  |  |  |  |  |  |  |  |  |
|  | **Post SCB** | 0.52 | 0.52 | 1.00 |  |  |  |  |  |  |  |  |  |  |  |  |  |  |  |
|  | **Pre SCB** | 0.51 | 0.52 | 1.00 | 1.00 |  |  |  |  |  |  |  |  |  |  |  |  |  |  |
|  | **PostUCB** | 0.50 | 0.50 | 0.96 | 0.96 | 1.00 |  |  |  |  |  |  |  |  |  |  |  |  |  |
|  | **Pre UCB** | 0.51 | 0.51 | 0.96 | 0.96 | 1.00 | 1.00 |  |  |  |  |  |  |  |  |  |  |  |  |
| **BMIQ** | **Post Adj** | -0.40 | -0.41 | -0.43 | -0.44 | -0.40 | -0.42 | 1.00 |  |  |  |  |  |  |  |  |  |  |  |
|  | **Pre Adj** | -0.40 | -0.41 | -0.42 | -0.43 | -0.38 | -0.40 | 0.97 | 1.00 |  |  |  |  |  |  |  |  |  |  |
|  | **Post SCB** | 0.10 | 0.10 | 0.47 | 0.46 | 0.48 | 0.46 | 0.07 | 0.09 | 1.00 |  |  |  |  |  |  |  |  |  |
|  | **Pre SCB** | 0.10 | 0.09 | 0.41 | 0.41 | 0.42 | 0.40 | 0.10 | 0.16 | 0.97 | 1.00 |  |  |  |  |  |  |  |  |
|  | **PostUCB** | 0.06 | 0.05 | 0.40 | 0.39 | 0.44 | 0.42 | 0.12 | 0.13 | 0.95 | 0.92 | 1.00 |  |  |  |  |  |  |  |
|  | **Pre UCB** | 0.03 | 0.02 | 0.31 | 0.31 | 0.35 | 0.33 | 0.12 | 0.19 | 0.91 | 0.94 | 0.96 | 1.00 |  |  |  |  |  |  |
| **SWAN** | **Post Adj** | 0.36 | 0.35 | 0.22 | 0.21 | 0.24 | 0.22 | 0.15 | 0.16 | 0.68 | 0.72 | 0.64 | 0.65 | 1.00 |  |  |  |  |  |
|  | **Pre Adj** | 0.40 | 0.39 | 0.26 | 0.25 | 0.28 | 0.26 | 0.12 | 0.13 | 0.68 | 0.71 | 0.63 | 0.64 | 0.99 | 1.00 |  |  |  |  |
|  | **Post SCB** | 0.13 | 0.12 | 0.41 | 0.40 | 0.39 | 0.37 | -0.05 | -0.02 | 0.90 | 0.90 | 0.84 | 0.84 | 0.77 | 0.75 | 1.00 |  |  |  |
|  | **Pre SCB** | 0.17 | 0.16 | 0.42 | 0.41 | 0.41 | 0.38 | -0.04 | 0.00 | 0.90 | 0.90 | 0.85 | 0.84 | 0.78 | 0.77 | 0.99 | 1.00 |  |  |
|  | **PostUCB** | 0.16 | 0.15 | 0.39 | 0.37 | 0.41 | 0.39 | -0.03 | 0.00 | 0.89 | 0.88 | 0.87 | 0.85 | 0.79 | 0.77 | 0.97 | 0.96 | 1.00 |  |
|  | **Pre UCB** | 0.18 | 0.17 | 0.39 | 0.37 | 0.42 | 0.40 | -0.03 | 0.00 | 0.88 | 0.87 | 0.87 | 0.85 | 0.79 | 0.78 | 0.96 | 0.96 | 1.00 | 1.00 |

**Table S6 Spearman Rank Correlations for p value rankings: Short-Haired in Tabby**

|  |  | **SQN** | | | | | | **BMIQ** | | | | | | **SWAN** | | | | | |
| --- | --- | --- | --- | --- | --- | --- | --- | --- | --- | --- | --- | --- | --- | --- | --- | --- | --- | --- | --- |
|  |  | **Post**  **Adj** | **Pre**  **Adj** | **Post**  **SCB** | **Pre**  **SCB** | **Post**  **UCB** | **Pre**  **UCB** | **Post**  **Adj** | **Pre**  **Adj** | **Post**  **SCB** | **Pre**  **SCB** | **Post**  **UCB** | **Pre**  **UCB** | **Post**  **Adj** | **Pre**  **Adj** | **Post**  **SCB** | **Pre**  **SCB** | **Post**  **UCB** | **Pre**  **UCB** |
| **SQN** | **Post Adj** | 1.00 |  |  |  |  |  |  |  |  |  |  |  |  |  |  |  |  |  |
|  | **Pre Adj** | 1.00 | 1.00 |  |  |  |  |  |  |  |  |  |  |  |  |  |  |  |  |
|  | **Post SCB** | 0.64 | 0.64 | 1.00 |  |  |  |  |  |  |  |  |  |  |  |  |  |  |  |
|  | **Pre SCB** | 0.64 | 0.64 | 1.00 | 1.00 |  |  |  |  |  |  |  |  |  |  |  |  |  |  |
|  | **PostUCB** | 0.57 | 0.58 | 0.96 | 0.96 | 1.00 |  |  |  |  |  |  |  |  |  |  |  |  |  |
|  | **Pre UCB** | 0.58 | 0.59 | 0.96 | 0.96 | 1.00 | 1.00 |  |  |  |  |  |  |  |  |  |  |  |  |
| **BMIQ** | **Post Adj** | 0.53 | 0.53 | -0.11 | -0.10 | -0.18 | -0.16 | 1.00 |  |  |  |  |  |  |  |  |  |  |  |
|  | **Pre Adj** | 0.50 | 0.50 | -0.13 | -0.13 | -0.20 | -0.18 | 0.98 | 1.00 |  |  |  |  |  |  |  |  |  |  |
|  | **Post SCB** | -0.02 | -0.02 | -0.17 | -0.17 | -0.23 | -0.23 | 0.47 | 0.46 | 1.00 |  |  |  |  |  |  |  |  |  |
|  | **Pre SCB** | -0.06 | -0.06 | -0.19 | -0.19 | -0.26 | -0.26 | 0.42 | 0.45 | 0.96 | 1.00 |  |  |  |  |  |  |  |  |
|  | **PostUCB** | -0.02 | -0.02 | -0.20 | -0.21 | -0.23 | -0.23 | 0.44 | 0.43 | 0.88 | 0.85 | 1.00 |  |  |  |  |  |  |  |
|  | **Pre UCB** | -0.08 | -0.08 | -0.25 | -0.26 | -0.28 | -0.29 | 0.42 | 0.44 | 0.86 | 0.88 | 0.97 | 1.00 |  |  |  |  |  |  |
| **SWAN** | **Post Adj** | 0.56 | 0.55 | -0.03 | -0.02 | -0.06 | -0.05 | 0.87 | 0.87 | 0.35 | 0.32 | 0.38 | 0.36 | 1.00 |  |  |  |  |  |
|  | **Pre Adj** | 0.58 | 0.57 | -0.02 | -0.01 | -0.06 | -0.05 | 0.89 | 0.88 | 0.37 | 0.34 | 0.39 | 0.37 | 0.99 | 1.00 |  |  |  |  |
|  | **Post SCB** | 0.11 | 0.11 | 0.21 | 0.21 | 0.16 | 0.15 | 0.09 | 0.10 | 0.60 | 0.60 | 0.54 | 0.55 | 0.29 | 0.28 | 1.00 |  |  |  |
|  | **Pre SCB** | 0.12 | 0.12 | 0.24 | 0.24 | 0.19 | 0.17 | 0.07 | 0.06 | 0.61 | 0.60 | 0.56 | 0.55 | 0.23 | 0.24 | 0.97 | 1.00 |  |  |
|  | **PostUCB** | 0.15 | 0.15 | 0.17 | 0.17 | 0.15 | 0.14 | 0.18 | 0.19 | 0.66 | 0.65 | 0.66 | 0.66 | 0.36 | 0.36 | 0.95 | 0.93 | 1.00 |  |
|  | **Pre UCB** | 0.14 | 0.14 | 0.20 | 0.19 | 0.17 | 0.15 | 0.16 | 0.14 | 0.67 | 0.65 | 0.68 | 0.67 | 0.30 | 0.32 | 0.94 | 0.96 | 0.97 | 1.00 |

**Table S7 Spearman Rank Correlations for p value ranks for Infant Sex**

|  |  | **SQN** | | | | | | **BMIQ** | | | | | | **SWAN** | | | | | |
| --- | --- | --- | --- | --- | --- | --- | --- | --- | --- | --- | --- | --- | --- | --- | --- | --- | --- | --- | --- |
|  |  | **Post**  **Adj** | **Pre**  **Adj** | **Post**  **SCB** | **Pre**  **SCB** | **Post**  **UCB** | **Pre**  **UCB** | **Post**  **Adj** | **Pre**  **Adj** | **Post**  **SCB** | **Pre**  **SCB** | **Post**  **UCB** | **Pre**  **UCB** | **Post**  **Adj** | **Pre**  **Adj** | **Post**  **SCB** | **Pre**  **SCB** | **Post**  **UCB** | **Pre**  **UCB** |
| **SQN** | **Post Adj** | 1.00 |  |  |  |  |  |  |  |  |  |  |  |  |  |  |  |  |  |
|  | **Pre Adj** | 0.99 | 1.00 |  |  |  |  |  |  |  |  |  |  |  |  |  |  |  |  |
|  | **Post SCB** | 0.98 | 0.98 | 1.00 |  |  |  |  |  |  |  |  |  |  |  |  |  |  |  |
|  | **Pre SCB** | 0.98 | 1.00 | 0.99 | 1.00 |  |  |  |  |  |  |  |  |  |  |  |  |  |  |
|  | **PostUCB** | 0.96 | 0.97 | 1.00 | 0.98 | 1.00 |  |  |  |  |  |  |  |  |  |  |  |  |  |
|  | **Pre UCB** | 0.95 | 0.98 | 0.99 | 0.99 | 0.99 | 1.00 |  |  |  |  |  |  |  |  |  |  |  |  |
| **BMIQ** | **Post Adj** | 0.51 | 0.44 | 0.55 | 0.46 | 0.55 | 0.51 | 1.00 |  |  |  |  |  |  |  |  |  |  |  |
|  | **Pre Adj** | 0.51 | 0.44 | 0.55 | 0.46 | 0.55 | 0.51 | 1.00 | 1.00 |  |  |  |  |  |  |  |  |  |  |
|  | **Post SCB** | 0.41 | 0.34 | 0.45 | 0.35 | 0.46 | 0.41 | 0.99 | 0.99 | 1.00 |  |  |  |  |  |  |  |  |  |
|  | **Pre SCB** | 0.41 | 0.34 | 0.45 | 0.35 | 0.46 | 0.41 | 0.99 | 0.99 | 1.00 | 1.00 |  |  |  |  |  |  |  |  |
|  | **PostUCB** | 0.50 | 0.43 | 0.54 | 0.45 | 0.54 | 0.50 | 0.98 | 0.98 | 0.98 | 0.98 | 1.00 |  |  |  |  |  |  |  |
|  | **Pre UCB** | 0.48 | 0.41 | 0.51 | 0.42 | 0.52 | 0.47 | 0.99 | 0.99 | 0.98 | 0.98 | 0.99 | 1.00 |  |  |  |  |  |  |
| **SWAN** | **Post Adj** | 0.53 | 0.48 | 0.57 | 0.48 | 0.58 | 0.54 | 0.96 | 0.96 | 0.94 | 0.94 | 0.94 | 0.94 | 1.00 |  |  |  |  |  |
|  | **Pre Adj** | 0.55 | 0.51 | 0.60 | 0.51 | 0.61 | 0.57 | 0.96 | 0.96 | 0.95 | 0.95 | 0.95 | 0.94 | 0.99 | 1.00 |  |  |  |  |
|  | **Post SCB** | 0.44 | 0.39 | 0.49 | 0.39 | 0.50 | 0.45 | 0.95 | 0.95 | 0.96 | 0.96 | 0.93 | 0.93 | 0.99 | 0.98 | 1.00 |  |  |  |
|  | **Pre SCB** | 0.47 | 0.43 | 0.52 | 0.43 | 0.54 | 0.49 | 0.95 | 0.95 | 0.96 | 0.96 | 0.94 | 0.93 | 0.98 | 0.99 | 0.99 | 1.00 |  |  |
|  | **PostUCB** | 0.51 | 0.46 | 0.56 | 0.47 | 0.58 | 0.53 | 0.95 | 0.95 | 0.94 | 0.94 | 0.95 | 0.95 | 0.98 | 0.99 | 0.98 | 0.98 | 1.00 |  |
|  | **Pre UCB** | 0.55 | 0.51 | 0.59 | 0.51 | 0.60 | 0.56 | 0.96 | 0.96 | 0.95 | 0.95 | 0.96 | 0.95 | 0.98 | 0.99 | 0.96 | 0.98 | 0.99 | 1.00 |
